# A key role for transketolase-like 1 in tumor metabolic reprogramming

**DOI:** 10.18632/oncotarget.10429

**Published:** 2016-07-06

**Authors:** Santiago Diaz-Moralli, Esther Aguilar, Silvia Marin, Johannes F. Coy, Mieke Dewerchin, Maciek R. Antoniewicz, Oscar Meca-Cortés, Leen Notebaert, Bart Ghesquière, Guy Eelen, Timothy M. Thomson, Peter Carmeliet, Marta Cascante

**Affiliations:** ^1^ Department of Biochemistry and Molecular Biomedicine, Faculty of Biology, Universitat de Barcelona, Barcelona, Spain; ^2^ Institute of Biomedicine of Universitat de Barcelona (IBUB) and CSIC-Associated Unit, Barcelona, Spain; ^3^ Tavargenix GmbH, Frankfurt am Main, Germany; ^4^ Zyagnum AG, Frankfurt am Main, Germany; ^5^ Laboratory of Angiogenesis and Vascular Metabolism, Department of Oncology, University of Leuven, Leuven, Belgium; ^6^ Laboratory of Angiogenesis and Vascular Metabolism, Vesalius Research Center, Leuven, Belgium; ^7^ Department of Chemical and Biomolecular Engineering, Metabolic Engineering and Systems Biology Laboratory, University of Delaware, Newark, DE, USA; ^8^ Department of Cell Biology, Institute for Molecular Biology of Barcelona, National Research Council (IBMB-CSIC), Barcelona, Spain

**Keywords:** tumor metabolism, metabolic reprogramming, transketolase-like 1, lipid metabolism, pentose phosphate pathway

## Abstract

Metabolic reprogramming, a crucial cancer hallmark, shifts metabolic pathways such as glycolysis, tricarboxylic acid cycle or lipogenesis, to enable the growth characteristics of cancer cells. Here, we provide evidence that transketolase-like 1 (TKTL1) orchestrates aerobic glycolysis, fatty acid and nucleic acid synthesis, glutamine metabolism, protection against oxidative stress and cell proliferation. Furthermore, silencing of TKTL1 reduced the levels of sphingolipids such as lactosylceramide (a sphingolipid regulating cell survival, proliferation and angiogenesis) and phosphatidylinositol (which activates PI3K/Akt/mTOR signaling). Thus, in addition to its well-known roles in glucose and amino acid metabolism, TKTL1 also regulates lipid metabolism. In conclusion, our study provides unprecedented evidence that TKTL1 plays central roles in major metabolic processes subject to reprogramming in cancer cells and thus identifies TKTL1 as a promising target for new anti-cancer therapies.

## INTRODUCTION

Because an evident feature of cancer cells is their accelerated proliferation rate, signals mediating the response to mitogenic stimuli and alterations in (proto-)oncogenes were among the first processes to be mechanistically addressed and identified in cancer. More recently, it has become evident that cancer cells must also undergo a metabolic reprogramming to sustain the abnormal growth characteristics associated with malignant transformation [1–3]. Consequently, different metabolic genes have been proposed to function as (proto-)oncogenes [4, 5]. Among them, glucose-6-phosphate dehydrogenase (*g6pd*) and transketolase-like 1 (*tktl1*) are of special interest because they regulate the pentose phosphate pathway (PPP), which generates NADPH for redox homeostasis and lipid synthesis and balances the flux of glucose derivatives between production of pentoses for nucleotide synthesis and supply of pyruvate for TCA cycle replenishment, or lactate production [6, 7].

Transketolase (TKT) is the key enzyme of the non-oxidative PPP branch (non-oxPPP). Via genome duplication and exon skipping, higher vertebrates acquired transketolase-like 1 (TKTL1), which compared to TKT protein lacks 38 amino acid residues [8, 9]. TKTL1 is upregulated in various cancer cells and tissues [4, 5, 10–13], and TKTL1 overexpression in cancer patients is correlated with malignancy, invasiveness, therapeutic resistance and poor prognosis [11, 14–16]. TKTL1 is required for rapid growth and viability of tumor cells [17], and protects against apoptosis induced by growth factor withdrawal [18], oxidative stress [17–19], cytotoxic therapy or targeted therapies [16, 20], while TKTL1 silencing hinders cancer cell proliferation [17, 21]. *In vivo*, silencing of TKTL1 in cancer cells reduces tumor growth, while overexpression of TKTL1 has opposite effects [7, 22]. However, little is known about how TKTL1 participates in all these processes that guarantee the growth and survival of cancer cells. Apart from the aforementioned effects, TKTL1 could also have a role in the reactions of the PPP. However, the precise reaction scheme of the non-oxPPP still remains debated, as the degree and distribution of ^14^C-isotope labeling differ from what has been predicted by the scheme of the individual reactions [23].

Unlike TKT, which catalyzes the common two-substrate reaction by using xylulose-5-phosphate (X5P) as donor and ribose-5-phosphate (R5P) as acceptor, it has been proposed that TKTL1 may perform a one-substrate reaction by converting X5P to glyceraldehyde-3-phosphate (GA3P) and acetate, similar to phosphoketolase, an enzyme that performs a similar one-substrate reaction in heterofermentative lactic acid bacteria [8, 24]. The acetate, generated in this reaction, can then be converted to acetyl-CoA (AcCoA).

Cancer cells often have increased rates of fatty acid synthesis and therefore require large amounts of AcCoA. However, the issue of the supply of AcCoA for fatty acid synthesis in glycolytic cancer cells is still unresolved, since AcCoA production from pyruvate in cancer cells is often reduced due to: i) a switch from pyruvate kinase (PK) M1 to the less active PKM2 that reduces pyruvate production [25, 26] and ii) a decreased entry of pyruvate into the TCA cycle due to inactivation of pyruvate dehydrogenase (PDH) [27], which can be compensated by the entry of pyruvate into the TCA cycle via the anaplerotic conversion to oxaloacetate by pyruvate carboxylase (PC) [28] or glutamine catabolism [29–31]. Given that, in cancer cells, the bulk of pyruvate is directed to lactate and the flux from glutamine to AcCoA is low, it is challenging to explain how the large amounts of AcCoA necessary for fatty acid synthesis are generated. We hypothesized that the one-substrate reaction catalyzed by TKTL1 may help to explain this enigma. Our findings support this hypothesis and further contribute to clarify additional mechanisms by which TKTL1 coordinates metabolic reprogramming and tumor growth.

## RESULTS

### TKTL1 contributes to overall cellular transketolase activity

**Figure 1:**
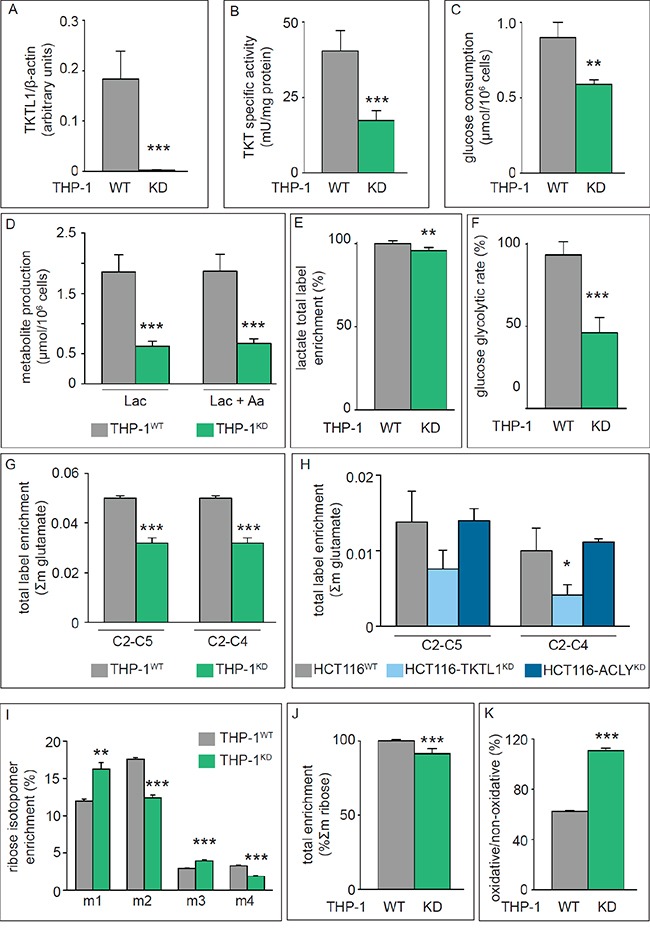
Effects of TKTL1 silencing on Transketolase activity, glycolysis, TCA Cycle and PPP **A.** Densitometric quantification of immunoblotting for TKTL1 in THP-1^WT^ and THP-1^KD^ cells. β-Actin was used as loading control (mean±SD; n=4; ***p<0.001). **B.** Enzymatic assay for total transketolase activity in THP-1^WT^ and THP-1^KD^ cells (mean±SD; n=8; ***p<0.001). **C, D.** Glucose consumption (C) and lactate and alanine production (D) in THP-1^WT^ and THP-1^KD^ cells (mean±SD; n=4; **p<0.01, ***p<0.001). **E.** Total label enrichment in lactate for THP-1^WT^ and THP-1^KD^ cells (mean±SD; n=4; **p<0.01; values for THP-1^WT^ were set to 100%). **F.** Glucose glycolytic rate in THP-1^WT^ and THP-1^KD^ cells (mean±SD; n=4; ***p<0.001). **G, H.** Label enrichment of fragments C2-C5 and C2-C4 of glutamate in THP-1^WT^ and THP-1^KD^ cells (mean±SD; n=6; ***p<0.001) (G) and in HCT116^WT^, HCT116-TKTL1^KD^ and HCT116-ACLY^KD^ cells (mean±SD; n=3; *p<0.05) (H). **I.** RNA ribose isotopologue distribution of ^13^C enrichment in THP-1^WT^ and THP-1^KD^ cells (mean±SD; n=3; **p<0.01, ***p<0.001). **J.** Total ^13^C RNA ribose enrichment calculated as Σm = m1+m2+m3+m4+m5 in THP-1^WT^ and THP-1^KD^ cells (mean±SD; n=5; ***p<0.001; values for THP-1^WT^ were set to 100%). **K.** Contribution of the oxPPP *versus* non-oxPPP, calculated as (m1/m2) (mean±SD; n=3; ***p<0.001). See also Figure 2 and Figure 4.

As an initial approach to characterize the relevance of TKTL1 in the metabolism and growth properties of cancer cells, we analyzed 4 cell lines with variable levels of TKTL1 (THP-1 and PC-3S, high expression; HCT-116 and PC-3M, low expression). Transduction of human THP-1 leukemia cells with a lentiviral vector expressing a TKTL1-specific shRNA (THP-1^KD^) specifically downregulated TKTL1 (Figure 1A) but not TKT (changed by 4.0 ± 1.2%; n = 3; p = NS). SiRNA-mediated silencing reduced TKTL1 expression by 86 ± 13% in human PC-3S prostate cancer cells, by 81 ± 13% in human PC-3M prostate cancer cells and by 60 ± 28% in human HCT116 colorectal carcinoma cells (n = 5; p < 0.01 for all). Consistent with the contribution by both TKT and TKTL1 to the overall transketolase activity according to their relative expression [10, 32], silencing of TKTL1 lowered but did not eliminate total transketolase activity (measured as GA3P production from R5P and X5P). Indeed, total transketolase activity was reduced by 60% in THP-1^KD^ cells (Figure 1B) and by 37% in PC-3S^KD^ cells (mU/mg protein: 8.0 ± 1.0 in PC-3S^WT^ cells *versus* 5.0 ± 0.4 in PC-3S^KD^ cells; n = 3; p < 0.05), but not in HCT116^KD^ or PC-3M^KD^ cells (changed by 7 ± 1% in HCT116^KD^ cells and by 1 ± 3% In PC-3M^KD^ cells; n = 5; p = ns), indicating that TKTL1 minimally contributed to the transketolase activity in the latter cells.

Proliferation of THP-1^KD^ cells was reduced by 21 ± 4% (n = 4; p < 0.005). In PC-3S^KD^ cells, TKTL1 silencing substantially affected viability, decreasing the cell population by 51 ± 12% (n = 6; p < 0.01) 96 hours after siRNA treatment. Interestingly, despite the minimal contribution of TKTL1 to the transketolase activity, proliferation was also reduced by 16 ± 5% (n = 6; p < 0.005) in HCT116^KD^ cells and by 21 ± 1% in PC-3M^KD^ cells (n = 3; p < 0.001), suggesting that TKTL1 affected cell proliferation independently of its transketolase activity. For the remainder of the study, we have used THP-1^KD^ and HCT116^KD^ cells as representative cells, in which TKTL1 did (THP-1^KD^) or did not (HCT116^KD^) contribute to total transketolase activity.

### The transketolase activity of TKTL1 drives glucose metabolism

Glucose consumption and lactate production were reduced by 34% and 66% in THP-1^KD^ cells (Figure 1C, 1D). Even when considering alanine synthesized from pyruvate, the total production of lactate plus alanine was reduced by 64% (Figure 1D). Furthermore, the lactate production *vs.* glucose consumption ratio was 1.1 ± 0.1 in THP-1^KD^ cells and 2.0 ± 0.4 in THP-1^WT^ cells, confirming that TKTL1 levels correlate with glucose metabolism and the Warburg effect [33]. [1,2-^13^C_2_]-glucose-based metabolic flux analysis confirmed that TKTL1 silencing reduced total lactate label enrichment (Figure 1E) and the glucose glycolytic rate (% of glucose converted to lactate and alanine, via glycolysis) by 45% in THP-1^KD^ cells (Figure 1F), indicating that the total amount and fraction of glucose consumed through glycolysis were reduced and that other uses of carbons from glucose were enhanced.

**Figure 2:**
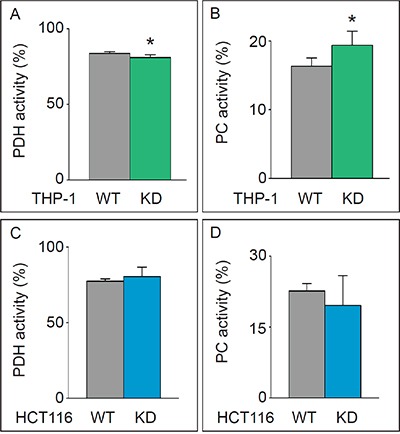
Analysis of glutamate enrichment **A, B.** Pyruvate dehydrogenase (PDH) (mean±SD; n=6; *p<0.05) (A) and pyruvate carboxylase (PC) activity (mean±SD; n=6; *p<0.05) (B) in THP-1^WT^ and THP-1^KD^ cells. **C, D.** Pyruvate dehydrogenase (PDH) (mean±SD; n=4; p=NS) (C) and pyruvate carboxylase (PC) activity (mean±SD; n=4; p=NS) (D) in HCT116^WT^ and HCT116-TKTL1^KD^ cells.

We measured the rate of glucose oxidation by analyzing the enrichment of [1,2-^13^C_2_]-glucose in two ^13^C-glutamate fragments, i.e. carbons 2 to 5 (C2-C5) and carbons 2 to 4 (C2-C4). Label incorporation into glutamate (Σm: glutamate enrichment) was reduced in THP-1^KD^ cells (Figure 1G). To estimate the role of PDH and pyruvate carboxylase (PC) in regulating the entry of glycolytic intermediates into the TCA cycle, we measured the PDH/PC ratio, whereby the PDH activity was measured as [m2(C2-C5) – m2(C2-C4)]/m2(C2-C5) and the PC activity as m2(C2-C4)/m2(C2-C5) [34]. Entry of pyruvate into the TCA cycle occurred primarily (80%) via the PDH pathway, but TKTL1 silencing did not, or only very modestly, affect the PDH/PC ratio in THP-1^KD^ cells (Figure 2A, 2B).

In HCT116^KD^ cells, in which TKTL1 did not significantly contribute to the overall transketolase activity, no differences in glucose consumption, lactate production (not shown), glucose oxidation (Figure 1H) or PDH/PC activities (Figure 2C, 2D) were observed. Thus, TKTL1 drives the glucose metabolism and contributes to the Warburg effect in cancer cells through its transketolase activity.

### TKTL1 contributes to the non-oxidative pentose phosphate pathway

**Figure 3:**
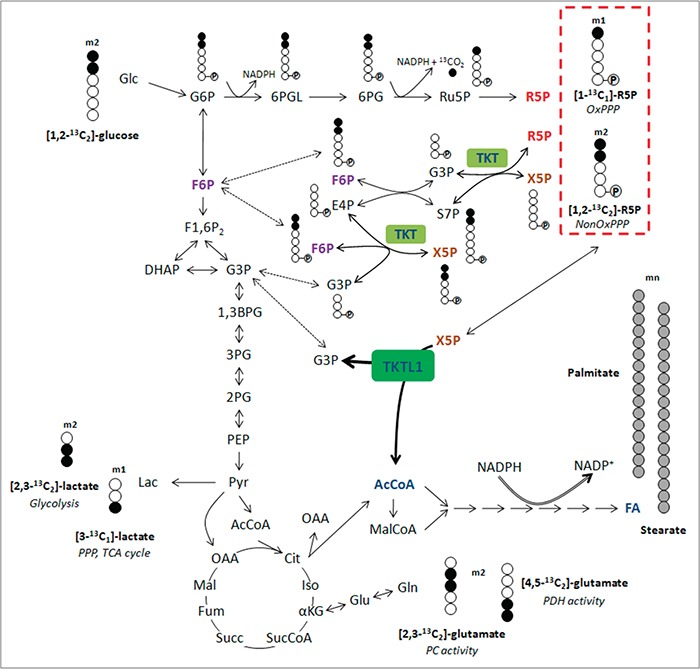
Overview of isotopomer formation through carbon distribution of [1,2-^13^C_2_]-glucose Distribution of carbon atoms from [1,2-^13^C_2_]-glucose results in the presence of different kinds of isotopomers from lactate, glutamate and ribose-5-phosphate and reflect the involvement of specific pathways (*italic*). Ribose-5-phosphate incorporating 3, 4 or 5 ^13^C atoms (m3, m4 and m5) is generated through recirculation of labeled molecules in the PPP. Carbons are represented by circles. Filled circles represent carbons that have incorporated label form [1,2-^13^C_2_]-glucose, open circles represent unlabeled carbons. Circles containing P represent phosphate groups. Abbreviations: m1, m2, isotopologues; 1,3BPG, 1,3-bisphosphoglycerate; 2PG, 2-phosphoglycerate; 3PG, 3-phosphoglycerate; 6PG, 6-phosphogluconate; 6PGL, 6-phosphogluconolactone; AcCoA, acetyl-CoA; Cit, citrate; DHAP, dihydroxyacetone phosphate; E4P, erythrose-4-phosphate; FA, fatty acids; F1,6P_2_, fructose-1,6-bisphosphate; F6P, fructose-6-phosphate; Fum, fumarate; G3P, glyceraldehyde-3-phosphate; Glc, glucose; G6P, glucose-6-phosphate; Iso, isocitrate; Lac, lactate; Mal, malate; MalCoA, malonyl-CoA; OAA, oxaloacetate; PEP, phosphoenolpyruvate; Pyr, pyruvate; R5P, ribose-5-phosphate; Ru5P, ribulose-5-phosphate; S7P, sedoheptulose-7-phosphate; Succ, succinate; SucCoA, succinyl-CoA; X5P, xylulose-5-phosphate; αKG, α-ketoglutarate.

**Figure 4:**
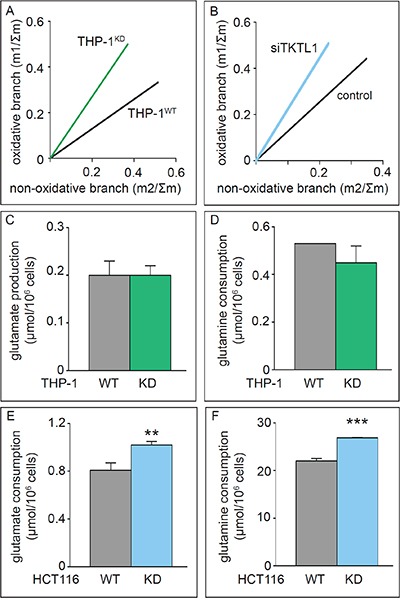
Effects of TKTL1 silencing on PPP unbalance and glutamine metabolism **A, B.** Phase plane analyses of ribose isotopologues m1 and m2 normalized to Σm in THP-1^WT^ and THP-1^KD^ cells (A) and of ribose isotopologues in HCT116 cells treated with control siRNA (control) and anti-TKTL1 siRNA (siTKTL1) (B). **C, D.** Glutamate accumulation (C) and glutamine consumption (D) in THP-1^WT^ and THP-1^KD^ cells (mean±SD; n=3; p=NS). **E, F.** Glutamate accumulation (E) and glutamine consumption (F) in HCT116^WT^ and HCT116-TKTL1^KD^ cells (mean±SD; n=3; **p<0.01, ***p<0.001).

We analyzed the role of TKTL1 in regulating the incorporation of glucose in the PPP by measuring metabolic fluxes via isotopomer analysis (Figure 3). After [1,2-^13^C_2_]-glucose incubation, the PPP flux was determined by measuring ribose enrichment: [1,2-^13^C_2_]-glucose generates m1 ribose if metabolized through the oxidative branch (oxPPP), whereas it produces m2 ribose via the non-oxPPP. Further recombination of labeled intermediates leads to m3 (oxPPP) and m4 (non-oxPPP) ribose. In THP-1^KD^ cells, TKTL1 silencing increased the pool of m1 and m3 ribose, while decreasing the pool of m2 and m4 ribose, indicating that the non-oxPPP flux was reduced (Figure 1I). Moreover, the decrease in total enrichment of ribose indicated that TKTL1 silencing reduced the net PPP flux by 10% (Figure 1J) and that the relative contribution of the oxPPP over the non-oxPPP flux, calculated as m1/m2, was increased in THP-1^KD^ cells (Figure 1K). Phase plane analysis of normalized m1 and m2 ribose isotopologues graphically illustrates these results (Figure 4A). Interestingly, similar results were obtained in HCT116^KD^ cells (Figure 4B), even though TKTL1 does not contribute to the total transketolase activity in these cells.

### TKTL1 silencing enhances glutamine consumption

In addition to glucose, glutamine is a major source of carbon atoms for the cell, anaplerotically replenishing the TCA cycle to maintain an active mitochondrial metabolism when pyruvate supply is compromised and enabling the production of AcCoA either through the TCA cycle or by forming citrate via reductive carboxylation [30, 31]. TKTL1 silencing in THP-1^KD^ cells did not affect glutamate accumulation or glutamine consumption (Figure 4C, 4D). In contrast, TKTL1 silencing in HCT116^KD^ cells increased glutamate and glutamine consumption (Figure 4E, 4F). However, even though glucose and glutamine metabolism were modulated differently in THP-1 and HCT116 cells, TKTL1 silencing induced similar changes in the ratio of glucose/glutamate consumption rates in both cell lines. This ratio was decreased by 19% in THP-1^KD^ cells and by 25% in HCT116^KD^ cells, indicating that TKTL1 regulates the balance between glucose and glutamine metabolism regardless of how it modulates each of these metabolic pathways individually or the extent of its contribution to overall transketolase activity.

**Figure 5:**
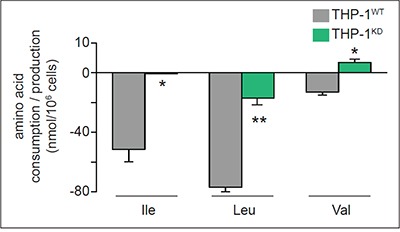
Impact of TKTL1 knock down on amino acid levels Amino acid (AA) levels in WT and TKTL1^KD^ THP-1 cells; positive values reflect amino acid production whereas negative values reflect AA consumption (mean±SD; n=3; *p<0.05, **p<0.01).

On the other hand, THP-1^KD^ cells reduced the consumption of isoleucine (Ile), leucine (Leu) and valine (Val), i.e. amino acids that are also catabolized to replenish the TCA cycle and generate AcCoA (Figure 5), indicating that TKTL1 favors these AcCoA production pathways.

### TKTL1 contributes to fatty acid synthesis

**Figure 6:**
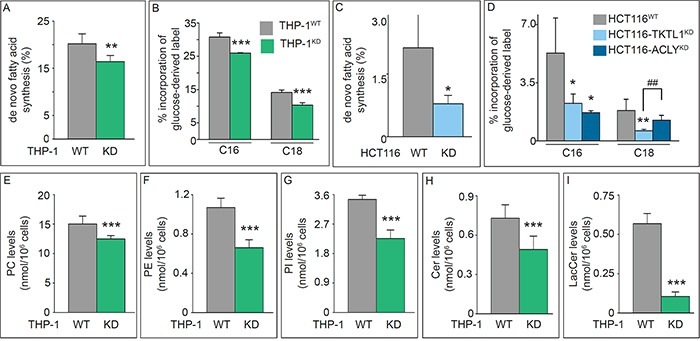
Effects of TKTL1 silencing on global cell lipidome **A, B.** Fraction of *de novo* synthesis of fatty acids (FNS) (mean±SD; n=6; **p<0.01) (A) and percentage of label incorporation from glucose into palmitate (C16) and stearate (C18) (mean±SD; n=5; ***p<0.001) (B) in THP-1^WT^ and THP-1^KD^ cells. **C, D.** Fraction of *de novo* synthesis of fatty acids (FNS) (mean±SD; n=4; *p<0.05) (C) and percentage of label incorporation from glucose into palmitate (C16) and stearate (C18) (mean±SD; n=4; *p<0.05, **p<0.01, ^##^p<0.01) (D) in HCT116^WT^, HCT116-TKTL1^KD^ and HCT116-ACLY^KD^ cells. **E-I.** Quantification of phospholipid (E-G) and sphingolipid (H, I) content in THP-1^WT^ and THP-1^KD^ cell lines by UPLC-MS (mean±SD; n=7; ***p<0.001). PC, phosphatidylcholine (E); PE, phosphatidylethanolamine (F); PI, phosphatidylinositol (G); Cer, ceramide (H); LacCer, lactosylceramide (I). See also Tables 2 and 3.

Given the importance of fatty acid synthesis in rapidly proliferating cells and the role of TKTL1 silencing on several pathways leading to AcCoA production, we analyzed whether TKTL1 silencing affects lipid synthesis. We determined [1,2-^13^C_2_]-glucose incorporation in C16-palmitate and C18-stearate as a surrogate read-out of [1,2-^13^C_2_]-glucose incorporation into AcCoA, and measured the fraction of *de novo* synthesized lipids (FNS) and the proportion of lipids synthesized by chain elongation. FNS levels and the contribution of [1,2-^13^C_2_]-glucose to fatty acid synthesis were reduced in THP-1^KD^ cells (Figure 6A, 6B), indicating that TKTL1 participated in the conversion of glucose carbon into fatty acids via generation of AcCoA. Similar effects were observed in HCT116^KD^ cells (Figure 6C, 6D). The decreased contribution of glucose to lipid synthesis was not attributable solely to a slower growth rate, since the incorporation of [1,2-^13^C_2_]-glucose into fatty acids was reduced more than the [1,2-^13^C_2_]-glucose incorporation into nucleotides (Table 1).

**Table 1:** Label incorporation from glucose into the fatty acids palmitate and stearate

Incorporation of glucose label into (ratio):	THP-1 cells		HCT116 cells	
	WT	KD	WT	KD
**palmitate *versus* nucleotides**	0.89 ± 0.01	0.82 ± 0.01***	0.17 ± 0.04	0.06 ± 0.02**
**stearate *versus* nucleotides**	0.40 ± 0.03	0.33 ± 0.02*	0.032 ± 0.004	0.017 ± 0.003**

Label incorporation of glucose in palmitate and stearate was compared to label incorporation in nucleotides in THP-1 and HCT116 cell (WT or silenced for TKTL1) (mean±SD; n=4; p-values are adjusted using a Holm method, *p<0.05, **p<0.01, ***p<0.005). The values represent the ratio of the incorporation of glucose label into palmitate/stearate *versus* incorporation of glucose label into nucleotides.

### TKTL1 contributes to the maintenance of the global cell lipidome

We also analyzed the lipidome by UPLC/MS and assigned > 200 lipid species: ~140 belonged to 11 groups of phospholipids (PL) (Table 2), of which phosphatidylcholines (PCs) (including the abundant PC 32:1 and PC 34:1 species), phosphatidylethanolamines (PEs), and phosphatidylinositols (PI) were reduced in THP-1^KD^ cells (Figure 6E-6G; Table 2). An additional 60 lipids were classified into 8 groups of sphingolipids (SL) (Table 3), of which ceramides (Cer) and lactosylceramides (LacCer) were reduced in THP-1^KD^ cells (Figure 6H, 6I). Thus, TKTL1 not only participates in lipid synthesis but also in maintaining the global cell lipidome.

**Table 2:** Effect of TKTL1 knock down on phospholipid synthesis in THP-1 cells

Species	Chain length (R)	THP-1^WT^	THP-1^KD^
**Phosphatidyl-cholines (PC)**	31:0	369 ± 24	438 ± 11.5
	32:2	285 ± 44	173 ± 29.8
	32:1	3533 ± 463	3008 ± 243
	32:0	1131 ± 67	888 ± 150
	33:3	64.1 ± 11.6	30.1 ± 4.1
	34:4	147 ± 20.9	172 ± 4.7
	34:4^●^	15.7 ± 2,8	9.33 ± 0.4
	34:3	53.3 ± 5.0	44.8 ± 8.3
	34:2	1422 ± 167	1249 ± 74.8
	34:1	3624 ± 861	3362 ± 604
	35:4	116 ± 18	55.8 ± 4.0
	35:2	99.7 ± 29.8	59.2 ± 8.4
	35:1	306 ± 45	208 ± 24.1
	36:6	9.64 ± 2.8	4.54 ± 1.2
	36:5	37.0 ± 9.0	20.0 ± 5.0
	36:4	147 ± 29	91.2 ± 14.4
	36:3	356 ± 53	236 ± 25
	36:2	1543 ± 187	1128 ± 241
	36:1	1282 ± 128	1302 ± 115
	37:0	5.24 ± 1.3	6.32 ± 2.6
	38:8	1.40 ± 0.6	0.74 ± 0.1
	38:7	4.67 ± 0.7	2.20 ± 0.2
	38:6	52.2 ± 17.5	33.0 ± 9.2
	38:5	101 ± 16	44.3 ± 2.5
	38:4	70.2 ± 11	48.7 ± 3.8
	38:4^●^	72.2 ± 7.6	93.9 ± 18.3
	38:3	97.8 ± 5.1	51.1 ± 16.0
	38:3^●^	34.2 ± 8.2	17.3 ± 3.7
	38:2	350 ± 114	120 ± 98.3
	38:0	8.33 ± 4.2	7.28 ± 1.4
	40:7	22.1 ± 10.9	11.1 ± 6.0
	40:6	9.94 ± 4.0	6.67 ± 1.9
	40:6^●^	12.7 ± 6.5	9.72 ± 1.7
	40:5	30.4 ± 17.8	17.0 ± 7.5
	40:2	27.1 ± 3.8	33.4 ± 3.4
	40:2^●^	59.6 ± 3.3	36.4 ± 5.0
	42:2	75.5 ± 5.3	49.4 ± 2.1
	42:2^●^	31.5 ± 4.1	22.7 ± 4.1
	44:3	17.7 ± 9.2	8.80 ± 2.7
	44:2	33.4 ± 24.1	9.99 ± 0.6
	44:2^●^	14.8 ± 9.0	13.1 ± 2.5
	44:1	64.5 ± 44.5	48.4 ± 7.7
	46:1	5.03 ± 3.1	4.48 ± 1.6
	**TOTAL**	**15033 ± 1319**	12481 ± 560 **
**Phosphatidyl-choline-plasmalogens (PC-pmg)**	34:2	16.7 ± 4.3	30.3 ± 6.5
	34:1	135 ± 12	171 ± 21
	34:0	1212 ± 139	1415 ± 137
	36:4	37.5 ± 7.5	36.6 ± 2.9
	36:3	11,7 ± 4.5	23.2 ± 3.0
	36:3^●^	33.1 ± 5.7	45.9 ± 7.9
	36:2	53.9 ± 0.7	25.6 ± 0.5
	36:2^●^	46.7 ± 5.0	-
	36:1	138 ± 5.7	77 ± 55
	36:1^●^	57.9 ± 13.5	14.8 ± 3.2
	36:0	263 ± 38	204 ± 97
	38:6	231 ± 33	179 ± 5.8
	38:5	29.9 ± 7.9	30.4 ± 5.8
	40:7	9.68 ± 2.6	6.00 ± 1.4
	40:6	18.4 ± 1.0	7.77 ± 1.8
	**TOTAL**	**1985 ± 255**	**2073 ± 454**
**Lyso-phospatidyl-cholines (LPC)**	16:1	204 ± 38	154 ± 23
	16:0	570 ± 154	579 ± 255
	18:3	100 ± 9	67.3 ± 13.6
	18:2	46.8 ± 6.6	39.9 ± 9.7
	18:1	352 ± 44	275 ± 39
	18:0	82.0 ± 14.6	124 ± 45
	20:4	85.0 ± 15.2	60.6 ± 16.7
	20:3	12.2 ± 0.8	15.2 ± 3.5
	20:1	19.5 ± 6.2	11.3 ± 2.3
	20:0	2.25 ± 0.1	1.79 ± 0.2
	**TOTAL**	**1373 ± 175**	**1246 ± 281**
**Lyso-phospatidyl-choline-plasmalogens (LPC-pmg)**	16:1	16.7 ± 4.9	7.93 ± 3.4
	16:0	80.6 ± 29.9	130.7 ± 41
	18.1	10.5 ± 2.4	12.3 ± 2.3
	18:0	10.4 ± 2.7	6.5 ± 1.2
	**TOTAL**	**109 ± 25**	153 ± 37 *
**Phosphatidyl-ethanolamines (PE)**	32:2	18.8 ± 7.3	11.9 ± 7
	32:1	125 ±25	88,2 ± 28.6
	32:0	7.49 ± 0,6	4.84 ± 1.0
	34:3	17.1 ± 6.1	9.98 ± 4.6
	34:2	126 ± 13	70.1 ± 10.2
	34:1	165 ± 21	126 ± 9.7
	36:5	40.7 ± 25.3	29.2 ± 3.1
	36:4	45.7 ± 7.7	26.9 ± 7.4
	36:3	79.4 ± 8.1	35.4 ± 10.1
	36:2	165 ± 29	84.5 ± 6.0
	36:1	100 ± 13	80.4 ± 8.8
	38:7	51.8 ± 3.7	43.9 ± 1.7
	38:6	31.2 ± 6.4	16.0 ± 2.3
	38:5	42.0 ± 11.9	20.4 ± 6.1
	38:4	65.6 ± 7.9	36.4 ± 6.7
	38:2	13.0 ± 2.6	7.3 ± 1.6
	38.1	3.63 ± 0.2	1.79 ± 0.1
	38:1^●^	13.5 ± 0.7	12.9 ± 0.5
	40:5	40.3 ± 9.4	24.7 ± 2.0
	**TOTAL**	**1069 ± 94**	660 ± 81 ***
**Phosphatidyl-ethanolamine-plasmalogens (PE-pmg)**	34:3	25.2 ± 2.9	19.9 ± 4.2
	34:2	267 ± 65	210 ±15
	34:1	354 ± 61	268 ± 35
	36:4	141 ± 36	131 ± 9
	36:4^●^	178 ± 5	159 ± 7
	36:3	67.9 ± 5.6	55.5 ± 2.9
	36:3^●^	69.2 ± 7.2	38.0 ± 2.3
	36:2	84.4 ± 49.1	47.5 ± 24.0
	36:2^●^	97.6 ± 13.9	46.0 ± 3.2
	36:1	102 ± 41	62.8 ± 6.2
	36:1^●^	100 ± 6	51.7 ± 3.2
	36:0	36.4 ± 9.8	26.0 ± 1.8
	38:6	72.9 ± 14.5	70.2 ± 1.9
	38:6^●^	113 ± 11	105 ± 5
	38:5	71.0 ± 11	84.4 ± 4.3
	38:5^●^	64.0 ± 1.9	57.8 ± 1.9
	38:4	37.3 ± 10.3	17.3 ± 2.1
	40:7	0.26 ± 0.45	0.73 ± 0.23
	40:6	7.22 ± 0.6	4.25 ± 0.2
	**TOTAL**	**1361 ± 220**	1030 ± 89 *
**Lyso-phosphatidyl-ethanolamines (LPE)**	16:1	18.8 ± 7.3	12.8 ± 2.6
	16:0	30.7 ± 8.1	27.6 ± 4.2
	18:2	15.6 ± 5.1	9.78 ± 1.5
	18:1	64.6 ± 24.7	36.7 ± 6.2
	18:0	44.4 ± 12.5	41.5 ± 4.1
	20:4	10.5 ± 3.2	6.25 ± 0.6
	20:3	8.1 ± 3.7	5.2 ± 2.3
	**TOTAL**	**193 ± 59**	140 ± 11 *
**Lyso-phosphatidyl-ethanolamine-plasmalogens (LPE-pmg)**	16:1	0.26 ± 0.07	0.24 ± 0.01
	16:0	84.7 ± 9.8	83.0 ± 4.4
	18:1	5.13 ± 1.7	2.98 ± 0.7
	18.0	22.3 ± 5.4	20.3 ± 1.5
	20:1	0.55 ± 0.04	0.44 ± 0.07
	20:0	1.76 ± 1.0	1.49 ± 0.4
	22:1	1.01 ± 0.1	0.74 ± 0.04
	22:0	1.13 ± 0.1	0.89 ± 0.07
	**TOTAL**	**66.8 ± 52.5**	**61.3 ± 45.4**
**Phospatidyl-serines (PS)**	32:1	34.0 ± 6.1	59.2 ± 1.4
	34:2	13.3 ± 1.7	14.5 ± 7.9
	34:1	157 ± 43	172 ± 98
	36:5	25.0 ± 0.6	22.8 ± 0.9
	36:2	77.7 ± 8.3	56.3 ± 28.7
	36:1	218 ± 26	167 ± 66
	38:3	41.3 ± 8.0	57.7 ± 23.0
	38:2	23.7 ± 1.8	7.16 ± 0.7
	38:1	18.2 ± 1.4	5.16 ± 0.2
	40:6	48.2 ± 5.5	54.1 ± 0.5
	40:3	3.26 ± 0.3	2.14 ± 0.3
	40:2	12.0 ± 0.6	3.02 ± 0.2
	40:1	31.8 ± 8.9	22.4 ± 14.1
	**TOTAL**	**621 ± 59**	**572 ± 276**
**Lyso-phospatidyl-serines (LPS)**	16:1	6.77 ± 1.6	12.8 ± 1.1
	16:0	3.35 ± 1.4	4.70 ± 3.6
	18:3	12.8 ± 3.8	9.93 ± 4.7
	18:2	27.2 ± 10.1	27.1 ± 17.0
	18:1	28.3 ± 7.0	24.5 ± 15.9
	18.0	60.2 ± 22.0	67.8 ± 45.7
	20:4	14.5 ± 3.7	11.0 ± 6.4
	20:3	43.7 ± 6.6	31.8 ± 15.7
	20:2	30.0 ± 4.3	34.3 ± 20.2
	22:1	0.65 ± 0.0	0.32 ± 0.19
	22:0	2.72 ± 0.23	0.14 ± 0.25
	**TOTAL**	**229 ± 51**	**220 ± 132**
**Phosphatidyl-inositols (PI)**	34:1	795 ± 178	708 ± 157
	34:0	47.0 ± 21.8	64.3 ± 32.1
	36:4	152 ± 54	187 ± 69
	36:3	128 ± 15	110 ± 20
	36:2	697 ± 45	347 ± 53
	36:1	804 ± 109	397 ± 34
	38:4	441 ± 119	259 ± 122
	38:3	306 ± 25	114 ± 11
	40:6	462 ± 24	279 ± 24
	**TOTAL**	**3452 ± 121**	2257 ± 262 ***

Values are expressed as pmol/10^6^ cells (mean±SD; n=7; p-values are adjusted using a Holm method, *p<0.05, **p<0.01, ***p<0.001). To calculate the totals ± standard deviations per lipid species, we first calculated per repeat the sums of all chain length values, and then calculated the average and standard deviation of these sums. Due to the fact that in this high-throughput analysis, a peak was not present for each chain length in every repeat, some totals listed do not equal the mathematical sum of the individual chain length values.

R, total number of carbons: total number of insaturated acyl moieties.

● Some species are detected by two independent peaks in the MS.

**Table 3:** Effect of TKTL1 knock down on sphingolipid synthesis in THP-1 cells

Species	Chain length (R)	THP-1^WT^	THP-1^KD^
**Ceramides (Cer)**	14:1	2.43 ± 0.3	0.89 ± 0.2
	14:0	8.40 ± 1.2	3.28 ± 1.1
	16:1	8.40 ± 1.5	2.91 ± 0.8
	16:0	158 ± 23	107 ± 40
	18:1	3.99 ± 0.3	1.47 ± 0.3
	18:0	20.8 ± 3.0	11.8 ± 5.2
	20:1	0.82 ± 0.12	0.30 ± 0.12
	20:0	4.67 ±0.4	2.59 ± 1.3
	22:1	4.52 ± 0.2	2.01 ±0.5
	22:1^●^	7.30 ± 1.0	2.71 ± 0.5
	22:0	40.9 ± 5.7	25.9 ± 6.3
	24:2	11.1 ± 1.6	3.14 ± 0.8
	24:1	63.6 ± 10.5	22.2 ± 6.6
	24:1^●^	32.6 ± 5.9	20.9 ± 2.4
	24:0	365 ± 85	285 ± 42
	**TOTAL**	**729 ± 103**	490 ± 103 **
**Dihydro-ceramides (DH-Cer)**	16:0	13.6 ± 2.6	6.46 ± 2.3
	18:0	2.98 ± 0.9	1.48 ± 1.4
	20:0	1.31 ± 0.2	0.78 ± 0.51
	22:0	12.0 ± 2.3	5.92 ± 2.5
	24:1	20.7 ± 11.4	12.4 ± 9.6
	24:0	60.8 ± 12.5	48.4 ± 28.5
	**TOTAL**	**123 ± 32**	75.4 ± 42.9 *
**Sphingomyelines (SM)**	14:1	4.15 ± 0.4	2.15 ± 0.6
	14:0	84.0 ± 25.7	55.9 ± 12.3
	16:1	89.3 ± 17.5	68.0 ± 8.6
	16:0	1783 ± 662	1776 ± 477
	18:1	38.6 ± 5.8	22.7 ± 3.8
	18:0^●^	140 ± 44	107 ± 16
	20:1	7.59 ±2.2	3.65 ± 1.5
	20:0	34.3 ± 6.9	24.6 ± 9.9
	22:2	3.57 ± 0.4	1.56 ± 0.4
	22:1	35.8 ± 2.5	21.3 ± 7.5
	22:1^●^	47.7 ± 7.0	26.9 ± 4.9
	22:0	170 ± 24	125 ± 32
	24:3	6.83 ± 1.5	2.85 ± 1.1
	24:2	67.6 ± 24.3	31.1 ± 12.2
	24:1	437 ± 122	195 ± 25
	24:1^●^	86.7 ± 22.5	72.1 ± 29.3
	24:0	665 ± 162	562 ± 71
	**TOTAL**	**3555 ± 432**	**3139 ± 474**
**Dihydro-sphingomyelines (DH-SM)**	14:0	11.6 ± 2.9	3.26 ± 0.7
	16:0	315 ± 104	76.8 ± 10.0
	18:0	26.7 ± 6.1	6.69 ± 2.6
	20:0	11.1 ± 2.9	3.87 ± 2.0
	22:0	50.2 ± 10.2	13.0 ± 4.8
	24:1	131 ± 46	23.9 ± 9.3
	24:0	201 ± 22	52.9 ± 14
	**TOTAL**	**728 ± 72**	166 ± 23 ***
**Glucosyl-ceramides (GlcCer)**	14:0	2.88 ± 0.5	2.80 ± 0.7
	16:0	71.4 ± 4.4	100 ± 8
	18:0	8.67 ± 2.1	8.45 ± 2.1
	20:0	2.21 ± 0.8	1.78 ± 0.8
	22:0	27.3 ± 8.3	30.9 ± 8.2
	24:1	18.5 ± 7.3	13.7 ± 5.2
	24:0	146 ± 27	281 ± 49
	**TOTAL**	**284 ± 41**	429 ± 67 **
**Glucosyl-dihydro-ceramides (GlcDH-Cer)**	16:0	17.7 ± 4.7	8.4 ± 2.6
	18:0	1.78 ± 0.4	0.66 ± 0.43
	20:0	1.45 ± 0.1	0.66 ± 0.43
	22:0	12.4 ± 1.4	4.63 ± 1.3
	24:0	56.5 ± 10.4	38.5 ± 7.6
	**TOTAL**	**87.9 ± 10.4**	57.1 ± 16.3 **
**Lactosyl-ceramides (LacCer)**	16:0	214 ± 19	33.5 ± 4.5
	18.0	23.3 ± 3.9	1.94 ± 0.5
	20:0	6.81 ± 0.7	0.65 ± 0.45
	22:0	53.6 ± 23.0	8.38 ± 5.5
	24:1	51.1 ± 23.0	3.74 ± 0.9
	24:0	224 ± 36	57.2 ± 18.0
	**TOTAL**	**569 ± 64**	105 ± 29 ***
**Lactosyl-dihydro-ceramides (LacDH-Cer)**	16:0	46.1 ± 10.0	1.31 ± 0.5
	18:0	5.32 ± 0.9	-
	20:0	2.47 ± 1.3	-
	22:0	25.7 ± 11.7	0.97 ± 0.77
	24:1	23.7 ± 11.7	3.49 ± 4.2
	24:0	91.8 ± 43.3	8.64 ± 4.3
	**TOTAL**	**195 ± 60**	14.4 ± 8.9 ***

Values are expressed as pmol/10^6^ cells (mean±SD; n=7; p-values are adjusted using a Holm method, *p<0.05, **p<0.01, ***p<0.001). To calculate the totals ± standard deviations per lipid species, we first calculated per repeat the sums of all chain length values, and then calculated the average and standard deviation of these sums. Due to the fact that in this high-throughput analysis, a peak was not present for each chain length in every repeat, some totals listed do not equal the mathematical sum of the individual chain length values.

R, total number of carbons:total number of insaturated acyl moieties.

● Some species are detected with two independent peaks in the MS.

### Evidence in support of one-substrate activity by TKTL1

**Figure 7:**
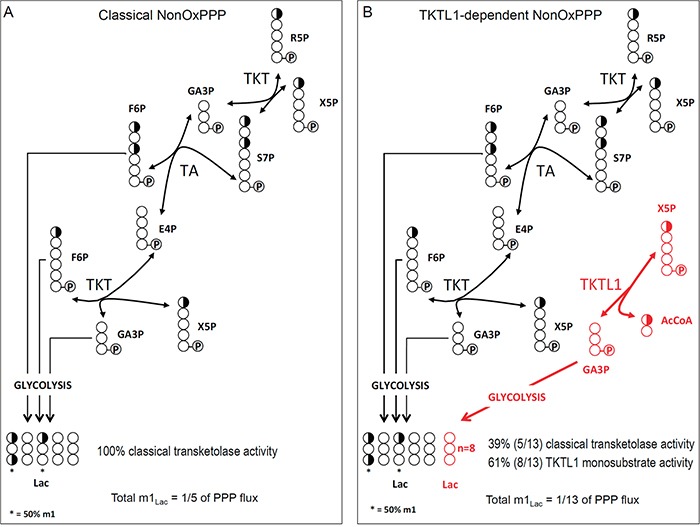
Label distribution depending on the mechanism of reaction of the TKTL1 Schematic representation of the classically accepted sequence of reactions of the non-oxPPP **A.**, and the proposed sequence of reactions for the non-oxPPP showing AcCoA production in the one-substrate reaction of TKTL1 (in red) **B.** Carbons are represented by circles. Half-filled circles represent carbons that have 50% chance of incorporating label from [1,2-^13^C_2_]-glucose; open circles represent unlabeled carbons. Circles containing P represent phosphate groups. R5P, ribose-5-phosphate; X5P, xylulose-5-phosphate; GA3P, glyceraldehyde-3-phosphate; S7P, sedoheptulose-7-phosphate; F6P, fructose-6-phosphate; E4P, erythrose-4-phosphate; AcCoA, acetyl-CoA; TKT, transketolase; TA, transaldolase; Lac, Lactate.

We explored if TKTL1 might display one-substrate activity by isotopologue analysis of m1 lactate, as this provides insight into the TKT/TKTL1-dependent glucose carbon flux through the PPP. We considered two possibilities: (i) both TKT and TKTL1 catalyze only the two-substrate reaction (Figure 7A), and (ii) TKT catalyzes the two-substrate reaction while TKTL1 catalyzes the one-substrate reaction (Figure 7B). To test this model, we analyzed the enrichment of [1,2-^13^C_2_]-glucose in m1 lactate, which can only be generated in the PPP via the two-substrate reaction, since the label is donated to AcCoA in the one-substrate reaction (Figure 4B, red pathway). Hence, in THP-1 cells, where TKTL1 accounts for 60% of the overall transketolase activity, TKTL1 silencing should *decrease* m1 lactate levels if it would only have the classical two-substrate activity (Figure 7A), but should *increase* m1 lactate levels if it had one-substrate activity (Figure 7B) since phosphorylated pentoses would be metabolized only via TKT generating more m1 lactate molecules.

This analysis revealed that the levels of m1 lactate were increased by 13 ± 7% (n = 6; p < 0.005) in THP-1^KD^ cells, which perfectly fits with theoretical predictions for the one-substrate reaction model (Figure 7B). In HCT116 cells, TKTL1 silencing decreased m1 lactate levels by 46 ± 10% (n = 4; p < 0.001). Given that TKTL1 minimally contributed to the overall transketolase activity in these cells, the effects on m1 lactate are unlikely to be due to its enzyme reaction but rather to the reduction of the non-oxPPP flux in TKTL1-silenced cells (Figure 4B).

### ACLY complements TKTL1 in the promotion of fatty acid synthesis

**Figure 8:**
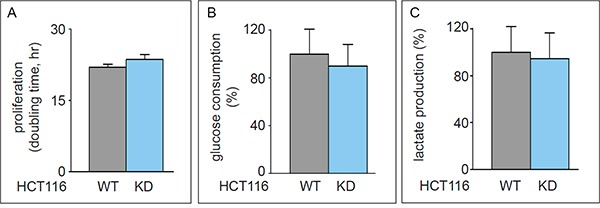
Effect of ACLY knock down in HCT116 cells **A.** Proliferation assay (mean±SD; n=3; p=0.21) in HCT116^WT^ and HCT116-ACLY^KD^. **B.** Glucose consumption (mean±SD; n=5; p=0.42). **C.** Lactate production (mean±SD; n=5; p=0.69) (mean±SD; n=5; p=0.40).

ATP-citrate lyase (ACLY) is a key enzyme regulating lipid synthesis by converting cytosolic citrate to AcCoA. To evaluate the possible role of TKTL1 in producing AcCoA in comparison with ACLY, we silenced ACLY in HCT116 (HCT116-ACLY^KD^) cells. SiRNA-mediated silencing reduced ACLY transcript levels by 45 ± 10% (n = 4; p < 0.001) without affecting proliferation, glucose consumption or lactate production (Figure 8A-8C), or altering the glycolytic rate or the PC/PDH ratio (not shown). ACLY silencing did not affect glutamate levels or glutamine consumption (not shown), nor did it alter glutamate enrichment (Figure 1H), but decreased fatty acid enrichment (Figure 6D), as expected. Indeed, when ACLY's activity is reduced, less citrate is processed to generate AcCoA and fewer labeled substrates are incorporated into palmitate without however affecting stearate enrichment (Figure 6D). These results, together with the abovementioned results in TKTL1 silenced cells, suggest that ACLY and TKTL1 play cooperative roles in fatty acids synthesis.

## DISCUSSION

In this study, we have found that TKTL1 plays broader roles in cell metabolism than previously anticipated. First, in agreement with previous data [7], we show that TKTL1 enhances glycolysis and sustains accelerated proliferation. These findings implicate TKTL1 in aerobic glycolysis (Warburg effect), the mechanism on which cancer and other proliferative cells rely to produce glycolytic intermediates for biomass synthesis and energy [25, 26, 35, 36]. TKTL1 also enhances the PPP flux for biosynthetic reactions, as the PPP generates ribose-5-phosphate for nucleic acid synthesis and NADPH for fatty acid synthesis and maintaining redox homeostasis to protect cells against apoptosis [37].

Second, TKTL1 affects glutamine metabolism, the other main carbon source in cancer cells. In contrast to its effects on glucose metabolism, we show that depletion of TKTL1 enhances glutamine consumption rates independent of its transketolase activity. Interestingly, the effect of TKTL1 on the ox-PPP *versus* non-ox PPP ratio is also independent of its contribution to the overall transketolase activity. Given that both glutaminolysis and PPP contribute to the maintenance of redox homeostasis, our results suggest that the previously described role of TKTL1 in protecting cells against oxidative stress [38] is independent of its contribution to the overall transketolase activity.

Although our results suggest that TKTL1 affects cell metabolism via mechanisms that are either dependent or independent of its enzymatic activity, they also unambiguously show that TKTL1 has transketolase enzymatic activity, supporting previous reports [10, 20, 32], and refuting studies claiming that TKTL1 lacks enzymatic activity [39, 40]. In our studies, TKTL1 silencing lowered the overall cellular transketolase activity, supporting that TKTL1 cleaves a two-carbon fragment from a pentose-phosphate to generate GA3P. Failure to detect *in vitro* transketolase activity of an artificial TKT protein variant with a 38 amino acid deletion was used in previous studies as an argument against an intrinsic enzymatic function of TKTL1, although no experiments were performed with the wild-type TKTL1 protein [39]. We speculate that the lack of transketolase activity by this TKT deletion variant might be due to defective interactions with other cellular components or cofactors that may be required for its enzymatic activity. Alternatively, why TKTL1 activity is detected *in vivo*, but not *in vitro*, might be attributed to the weak binding of TKTL1 to its cofactor, thiamine diphosphate, which retains the two-carbon fragment upon cleavage of X5P until it is transferred to R5P.

Our characterization of the oncogenic role of TKTL1 also sheds light on two largely unexplained phenomena in cancer cell metabolism. First, the TKTL1 activity described here better reconciles with the proposed hypothetical reaction scheme [23]. Second, our observations may help resolve the outstanding enigma of how rapidly proliferating cells can boost lipid synthesis [41] when AcCoA production is limited by impaired pyruvate production from phosphoenolpyruvate (because of PKM2 overexpression) [25, 26] and inhibition of PDH (which drives pyruvate to lactate, thereby reducing pyruvate entry into the TCA cycle) [27, 42, 43]. Taking into account that i) silencing of TKTL1 or ACLY in HCT116 cells reduced the expression of the corresponding protein to a similar extent (around 50%), and ii) the decrease in palmitate enrichment from labeled glucose was similar for both knock downs (around 40-60%), it can be estimated that a fully active TKTL1 can contribute to up to 50% of the flux from glucose to fatty acid synthesis. Indeed, our study shows that TKTL1 plays a significant role in the synthesis of fatty acids and phospholipids.

The observed enrichment of [1,2-^13^C_2_]-glucose in m1 lactate is consistent with the one-substrate reaction previously suggested by Coy *et al*. [8]. In this regard, the 38-amino acid segment in TKT missing in TKTL1 contains a critical histidine residue, homologous to His103 in the *S. cerevisiae* transketolase protein. Site-directed mutagenesis of this residue in the yeast transketolase converts its activity from the classical two-substrate reaction to a one-substrate reaction [44]. We speculate that, by analogy, the absence of the equivalent His residue in mammalian TKTL1 may favor the one-substrate reaction, whereby X5P is converted to GA3P and a two-carbon fragment. This fragment could be AcCoA or acetate (that subsequently generates AcCoA via acetyl-CoA synthetase 2) [45, 46]. Thus, TKTL1 may link the synthesis of nucleotide precursors (R5P) to the production of fatty acid precursors (AcCoA), providing an alternative explanation for how cancer cells can generate new lipids when AcCoA production from glucose metabolism is restricted. This TKTL1-specific one-substrate reaction would also explain why TKTL1 silencing in HCT116^KD^ and PC-3M^KD^ cells reduces cell proliferation, while not affecting overall transketolase activity. We thus speculate that the evolutionary removal in TKTL1 of the 38 amino acid residues present in TKT, which includes the His residue equivalent to yeast His103, has created a new enzyme capable of catalyzing a one-substrate reaction that enables the fermentation of glucose to lactic acid and AcCoA even in the presence of oxygen.

Our study therefore shows that TKTL1 is an active transketolase that modulates the Warburg effect to provide energy and precursors for DNA replication and membrane biogenesis depending on its enzymatic activity. Moreover, our results suggest that TKTL1 controls redox homeostasis and protects cells against oxidative stress and apoptosis independent of its transketolase activity. Finally, we have shown that TKTL1 participates in the synthesis of lactosylceramides, a sphingolipid regulating cell survival, proliferation and angiogenesis [47, 48], and phosphatidylinositols, which activate PI3K/Akt/mTOR signaling [49].

In conclusion, we provide unprecedented evidence that TKTL1 confers a growth advantage to proliferating cells and is essential in cancer metabolic reprogramming, suggesting that TKTL1 might be a promising target for the design of future anti-cancer therapies.

## MATERIALS AND METHODS

### Chemicals and reagents

All chemicals were purchased from Sigma-Aldrich Co (St Louis, MO, USA), unless otherwise specified. Antibiotics (10,000 U/ml penicillin, 10,000 μg/ml streptomycin) were obtained from Gibco-BRL (Eggenstein, Germany), fetal calf serum (FCS) and trypsin-EDTA solution C (0.05% trypsin - 0.02% EDTA) from PAA Laboratories (Pasching, Austria). Stable [1,2-^13^C_2_]-D-glucose isotope was obtained with > 99% purity and 99% isotope enrichment for each carbon from Isotec Inc. (Miamisburg, OH). TRIzol™ reagent was obtained from Invitrogen (Paisley, UK). RNase was obtained from Roche Diagnostics (Mannheim, Germany). Phospholipid and sphingolipid standards were purchased from Sigma-Aldrich Co. (St. Louis, MO) (PC 34:0, PE 34:0, PG 34:0, PI 34:2, PS 34:0, LysoPC 17:0, Cer 12:0, GlcCer 12:0, LacCer 12:0 and SM 12:0) or Avanti Polar Lipids, Inc. (Alabaster, AL) (Cer 24:1 and sphinganine 17:0).

### Cell culture

Human THP-1 acute monocytic leukemia and human HCT116 colon carcinoma cells were obtained from the American Type Culture Collection (Manassas, VA, US). THP-1 WT and TKTL1^KD^ cells were generated by Sirion Biotech GmbH (Munich, Germany). THP-1 and HCT116 cell lines were grown in RPMI 1640 culture medium supplemented with 10% heat-inactivated FCS and antibiotics: 100 U/ml penicillin and 100 μg/ml. HCT116 were grown in DMEM:HAM F12 (1:1), supplemented with 10% heat-inactivated FCS, 2 mM L-glutamine, 1 mM pyruvic acid 1% non-essential amino acids, 100 U/ml penicillin and 100 μg/ml streptomycin. PC-3M and PC-3S human prostatic adenocarcinoma sub-lines were generated as described in [50] and were grown in RPMI 1640 medium supplemented with 10% heat-inactivated FCS, 1 mM pyruvic acid, 1% non-essential amino acids, 100 U/ml penicillin, 100 μg/ml streptomycin and 200 μg/ml geneticin.

### RT-PCR

Total RNA was isolated from cultured cells (approximately 1-2 × 10^6^ cells) using the TRIzol reagent (Roche Diagnostics). For quantitative RT-PCR, cDNA was synthesized from 1 μg of total RNA using random hexamers and M-MLV-reverse transcriptase (Invitrogen) and subjected to real-time PCR with specific TaqMan probes (Assays-on-demand TKTL1: Hs00202061_m1; ACLY: Hs00153764_m1; reference probe, PPIA: Hs99999904_m1) (Applied Biosystems, Foster City, CA) on a ABI Prism 7700 Sequence Detector System (Applied Biosystems). All reactions were performed in triplicate. Fold changes in gene expression were calculated using the ΔΔCt method, using as PPIA as a reference.

### Silencing of TKTL1 and ACLY

Human TKTL1 and ACLY-specific siRNAs and non-targeting control duplexes (Thermo Scientific Dharmacon, Lafayette, CO, USA) were transfected using Metafectene (Biontex, Munich, Germany). The highest efficiency in TKTL1 silencing was accomplished with 5′-GGAGUUGCAUGUGGAAUGG-3′ (catalog no. J-004736-06). For ACLY silencing a mixture of 4 siRNA was most effective (ON-TARGETplus SMARTpool: 5′-GCACGAAGUCACAAUCUUU-3′, 5′-CGAGUGAAGUCGAUAAACA-3′, 5′-GAGAGCA AUUCGAGAUUAC-3′ and 5′-CCACUCCUCUGCUC GAUUA-3′, catalog no. L-004915-00). RNA and protein expression assays were determined 72 and 96 h after transfection, respectively. Glucose consumption, lactate production and glutamate/glutamine variations were assessed 96 h post transfection. Transketolase activity was determined 96 and 120 h post transfection.

### Western blotting

Western blotting analyses were performed on cellular lysates (100 μl of lysis buffer was added per 10^6^ cells, lysis buffer contained 20 mM Tris HCl pH 7.5, 1 mM dithiothreitol (DTT), 1 mM EDTA, 0.2 g/L Triton X-100, 0.2 g/L sodium deoxycholate, 0.2 mM PMSF (phenylmethylsulfonyl fluoride) and 1/1000 orthovanadate, 1/500 PMSF, 1/100 leupeptin and 1/100 aprotinin. Following addition of the lysis buffer, cells were sonicated for 5 min in ice-water bath at low intensity (40 kHz) and centrifuged at 4°C for 10 min at 20,000 x g). Samples (30 μg protein) were resolved by 12% SDS-PAGE, transferred to PVDF membranes (Bio-Rad, El Prat de Llobregat, Spain), membranes were blocked with 5% milk in PBS for 1 h at 20°C and incubated overnight at 4°C with primary antibodies diluted in Tris-buffered saline containing 0.1% Tween 20 (TBS-T). After washing, membranes were incubated with the secondary antibody for 1 h at 20°C. Visualization of specific bands was accomplished by chemiluminescence (Millipore, Billerica, MA) in a LAS-3000 imaging system (Fuji Photo Film, Tilburg, The Netherlands). Antibodies used were: mouse monoclonal anti-TKTL1 antibody (clone JFC12T10), rabbit anti-TKT antibody, rabbit polyclonal anti-ACLY antibody (catalogue no. 4332, Cell Signaling Technology). Loading control was performed by Western blotting analysis for actin using mouse monoclonal (MP Biomedicals, Santa Ana, CA) or rabbit polyclonal (Sigma-Aldrich) anti-actin antibodies.

### Cell proliferation

Cells were seeded in 6-well plates at a concentration of 75,000 cells per well and counted using a Scepter Handheld Automated Cell Counter (Millipore). THP-1^WT^ and THP-1^KD^ cells were seeded in 75 cm^2^ flasks at a concentration of 200 × 10^3^ cells/mL in a total volume of 15 mL medium. Every 24 h until 120 h an aliquot of 1 mL medium was removed and cells were counted. Duplication times were determined as the slope of the graph obtained by plotting the napierian logarithm of cell concentration (cells/mL) versus the incubation time in hours corresponding to the period of cellular exponential growth.

### TKT enzymatic activity

Fresh cell pellets containing approximately 5-10 × 10^6^ cells were resuspended in lysis buffer (20 mM HEPES, pH 7.5, 10% glycerol, 0.4 mM NaCl, 10 mM EGTA, 5 mM EDTA, 0.4% Triton X-100, 25 mM NaF, 1 mM DTT, 0.4 mM Pefabloc SC, 20 μg/mL pepstatin), sonicated in ice-cold buffer (4°C) and immediately followed by centrifugation at 15,000 × g for 15 min at 4°C. The supernatant was used for the determination of enzyme activity with a Cobas Mira Plus analyzer (HORIBA ABX, Montpellier, France). Transketolase activity was determined as described [51]. Briefly, the TKT activity product, glyceraldehyde-3-phosphate, is isomerized to dihydroxyacetone-phosphate and its subsequent conversion to glycerol-phosphate produces 1 molecule of NADH that absorbs at 340 nm. Initially, 40 μL of sample were added to a cuvette containing 150 μL of buffer (50 mM Tris-HCl buffer, pH 7.6; 5 mM MgCl_2_; 0.1 mM thiamine pyrophosphate (TPP); 0.2 mM NADH; 0.2 U/mL triose phosphate isomerase and 0.2 U/mL glycerol dehydrogenase). The reaction mixture was kept at 37°C for 15 min in order to convert the trioses-phosphate into glycerol-phosphate. Finally, 75 μL of a solution containing 25 mM R5P and 25 mM X5P was transferred to the cuvette and absorbance at 340 nm was recorded for 5 min.

### Metabolite measurement

Glucose, lactate, glutamine and glutamate concentrations in media were determined as described [52, 53] using a Cobas Mira Plus instrument (Roche). Glutamine quantification was achieved by converting it to glutamate using glutaminase (Sigma-Aldrich). The isotopologue distribution of glucose, lactate, RNA ribose, glutamate and fatty acids after incubation with [1,2-^13^C_2_]-D-glucose (Isotec) was analyzed by GC-MS as described [54, 55] using a Shimadzu GCMS-QP2010 system (Duisburg, Germany).

### Amino acid quantification

Amino acid concentrations in cell culture medium were determined by ion-exchange chromatography with a Biochrom 30 amino acid analyzer (Pharmacia Biochrom Ltd, Cambridge, UK). 70 μL of 150 μM norleucine was used as an internal standard and added to 500 μL of medium. Solvent was evaporated to complete dryness using a Savant SpeedVac (Thermo Scientific, Waltham, MA, USA). The samples were redissolved in 500 μL lithium citrate buffer (pH 2.2), filtered through a 0.22 μm filter and 30 μL per sample injected onto the Biochrom 30 lithium system. A set of lithium citrate buffers were used as a mobile phase for separation for 115 min and post column derivatization with ninhydrin allowed amino acid quantification at 570 and 440 nm.

### Isotopologue analysis by GC-MS

For isotopologue distribution analysis, 3 × 10^5^ cells/mL were seeded in 75 cm^2^ Petri dishes in the presence of RPMI 1640 medium spiked with 10 mM of [1,2-^13^C_2_]-glucose (Sigma-Aldrich) (50% isotope enrichment), for 48 h. Following incubation, cells were counted with a Scepter Handheld Automated Cell Counter (Millipore) and centrifuged (1,500 rpm for 5 min). Medium was collected for glucose, lactate and glutamate/glutamine analysis, and cell pellets were frozen for RNA ribose and fatty acids analysis.

*Glucose* was isolated from 200 μL medium by ion-exchange chromatography and converted to its aldonitrile acetate derivative by adding 100 μL hydroxylamine in pyridine (2% wt) at 100°C for 30 min followed by 75 μL acetic anhydride at 100°C for 1 h. The reaction mixture was evaporated to complete dryness and redissolved in 50 to 200 μL of ethyl acetate. 1 μL of the derivatized product was loaded to a GC-2010 gas chromatograph (Shimadzu) equipped with a HP-5MS capillary column (30 m length, 250 μm diameter and 0.25 μm film thickness), connected to a GCMSQP2010 mass selective detector (Shimadzu) [54]. *Lactate* was extracted from the medium by ethyl acetate after acidification with HCl and 1 mL ethyl acetate at 20°C. The propylamideheptafluorobutyric derivative of the lactate was prepared and analyzed as described [55]. *RNA ribose* was isolated by acid hydrolysis of cellular RNA after Trizol (Invitrogen) extraction from cell pellets. Ribose was derivatized to its aldonitrile acetate form by an analogous derivatization procedure as described for glucose. After derivatization, the reaction mixture was evaporated to complete dryness and redissolved in 50 to 200 μL of ethyl acetate. *Glutamate* was isolated from 1 mL of cell medium using ion-exchange chromatography and converted to its n-trifluoroacetyl-n-butyl derivative by adding 200 μL of butanolic HCl (0.4 N), the mixture incubated at 100°C for 1 h and dried under nitrogen stream. Next, 100 μL methylene chloride and 25 μL trifluoroacetic acid were added. The solution was kept at 20°C for 20 min and dried under nitrogen stream. The pellet was dissolved in 50 to 200 μL methylene chloride and 1 μL was injected for analysis [34, 55]. *Fatty acids* were extracted with methanol from the organic phase obtained during RNA isolation with Trizol (Invitrogen). Purified fatty acids were converted to their methyl ester derivatives by adding 200 to 500 μL of methanolic HCl 0.5 N, heating at 70°C for 1 h, drying under nitrogen stream and dissolving in 200 μL of hexane for injecting 1 μL in the GC-MS, now equipped with a bpx70 (SGE) column (30 m length, 250 μm diameter and 0.25 μm film thickness).

### Lipidomic analysis

Two standard mixes of phospholipids (PL) and sphingolipids (SL) were prepared. The PL mix contained PC 34:0, PE 34:0, PG 34:0, PI 34:2, PS 34:0 and LysoPC 17:0 at a concentration of 20 μM each. The SL mixture contained Cer 12:0, GlcCer 12:0, LacCer 12:0, SM 12:0 and sphinganine 17:0 at a concentration of 20 μM each. These mixes were used as internal standards for PL and SL identification and quantification at a final concentration of 200 pmol/sample. Two pellets containing 750 × 10^3^ cells each were re-suspended in 100 μL of milliQ water and 500 μL pure methanol (MeOH), these suspensions were transferred to glass vials. 250 μL chloroform (CHCl_3_) was added to obtain an extraction solvent MeOH:CHCl_3_:H_2_O (2:1:0.4). 10 μL of PL or SL standard mix was added and lipids were extracted overnight at 48°C. For sphingolipid analysis, 75 μL of 1 M potassium hydroxide (KOH) dissolved in MeOH was added and samples were sonicated. After 2 h of incubation at 37°C, 75 μL of 1 M acetic acid was added. Following extraction, PL and SL samples were dried under nitrogen stream and stored at −20°C until further usage. Prior to analysis on an UPLC-MS, samples were dissolved in 500 μL MeOH and transferred to Eppendorf tubes. The volume was reduced by evaporation under gentle nitrogen steam to a final volume of 150 μL MeOH, prior to analysis samples were centrifuged at 10,000 x g for 3 min and 150 μL of supernatant was infused into the UPLC. The liquid chromatography–mass spectrometer (Waters Acquity UPLC system, connected to a Waters LCT Premier orthogonal accelerated time of flight mass spectrometer; Waters, Millford, MA) was operated in positive electrospray ionisation mode. Full scan spectra from 50 to 1,500 Da were acquired and individual spectra were summed to produce data points every 0.2 s. Mass accuracy and reproducibility were maintained by using an independent reference spray via LockSpray interference. The analytical column, maintained at 30°C, was a 100 × 2.1 mm id, 1.7 μm C8 Acquity UPLC BEH (Waters). The mobile phase consisted of solvent A: MeOH/H_2_O/HCOOH (74:25:1 v:v:v); and solvent B: MeOH/HCOOH (99:1 v:v), both containing 5 mM ammonium formate. A gradient was programmed - 0.0 min, 80% B; 3 min, 90% B; 6 min, 90% B; 15 min, 99% B; 18 min, 99% B; 20 min, 80% B; 22 min, 80% B, with a 300 μL/min flow rate. Quantification was carried out using the ion chromatogram extracted for each compound in 50 mDa windows. The linear dynamic range was determined by injecting standard mixtures. Positive identification of compounds was based on the accurate mass measurement with an error < 5 ppm and its LC retention time, compared to that of a standard (± 2%). Lipid species were assigned from the m/z values detected in the MS using the MassLynx software (v4.1 SCN627 Waters). Positive ionization allowed detection of PC, PC-pmg, Lyso-PC and Lyso-PC-pmg from the phospholipid extract and Cer, dhihydroceramides, SM, dihydrosphingomyelins, GlcCer, glucosyldihydroceramides, LacCer and lactosyldihydroceramides from the sphingolipid extract. Negative ionization mode allowed detecting the rest of phospholipid classes: PE, PE-pmg, Lyso-PE, Lyso-PE-pmg, PS, Lyso-PS and PI.

### Statistical analysis

*In vitro* experiments were performed in triplicate for each treatment in at least two independent experiments. Mass spectrometric analyses were carried out in three independent 1 μL injections of each sample by the automatic sampler in a linear set-up. Raw data were considered valid only if the sample deviation was < 1% of the normalized peak intensity. Statistical analyses were performed using the parametric unpaired, two-tailed independent sample Student's *t*-test, assuming normality of distribution and homogeneity of variance based on past experience in similar measurements [56, 57]. When a family of statistical hypotheses was tested in the same series of experiments, the Holm method for P-value adjustment was used [58]. *P* < 0.05 (*) was considered statistically significant.
